# TNF overexpression and dexamethasone treatment impair chondrogenesis and bone growth in an additive manner

**DOI:** 10.1038/s41598-022-22734-8

**Published:** 2022-10-28

**Authors:** Yunhan Zhao, Bettina Celvin, Maria C. Denis, Niki Karagianni, Cecilia Aulin, Farasat Zaman, Lars Sävendahl

**Affiliations:** 1 Department of Women’s and Children´s Health, Karolinska Institutet and Pediatric Endocrinology Unit, Karolinska University Hospital, J9:30, Visionsgatan 4, 171 64 Solna, Sweden; 2 Biomedcode Hellas S.A., 34 Al. Fleming Str, 16672 Vari, Greece; 3 Department of Medicine Solna, Karolinska Institutet and Division of Rheumatology, Karolinska University Hospital, Center for Molecular Medicine, Visionsgatan 18, 171 76 Solna, Sweden

**Keywords:** Rheumatology, Diseases, Medical research

## Abstract

Children with chronic inflammation are often treated with glucocorticoids (GCs) and many of them experience growth retardation. It is poorly understood how GCs interact with inflammatory cytokines causing growth failure as earlier experimental studies have been performed in healthy animals. To address this gap of knowledge, we used a transgenic mouse model where human TNF is overexpressed (huTNFTg) leading to chronic polyarthritis starting from the first week of age. Our results showed that femur bone length and growth plate height were significantly decreased in huTNFTg mice compared to wild type animals. In the growth plates of huTNFTg mice, increased apoptosis, suppressed Indian hedgehog, decreased hypertrophy, and disorganized chondrocyte columns were observed. Interestingly, the GC dexamethasone further impaired bone growth, accelerated chondrocyte apoptosis and reduced the number of chondrocyte columns in huTNFTg mice. We conclude that TNF and dexamethasone separately suppress chondrogenesis and bone growth when studied in an animal model of chronic inflammation. Our data give a possible mechanistic explanation to the commonly observed growth retardation in children with chronic inflammatory diseases treated with GCs.

## Introduction

Growth retardation affects many children with chronic inflammatory conditions, such as juvenile idiopathic arthritis (JIA), and many of these patients are treated with glucocorticoids (GCs)^1^. JIA is characterized by increased systemic levels of pro-inflammatory cytokines, such as TNF and IL-6, not only affecting the joints but also the growth plate cartilage causing growth retardation in many children^2^. Although clinical studies of chronic inflammation in childhood show that long-term treatment with GCs may lead to irreversible growth impairment^2,3^, the underlying mechanisms are still unclear.

Longitudinal bone growth occurs at the growth plate, a hyaline cartilage plate situated in the metaphysis at each end of long bones. Any disturbance within the growth plate cartilage may negatively affect longitudinal bone growth. TNF is a key player in chronic inflammation triggering other pro-inflammatory cytokines such as IL-1 and IL-6^4^. Locally, TNF and IL-1β synergistically cause growth retardation as confirmed in cultured fetal rat metatarsal bones by inhibiting chondrocyte proliferation and differentiation while inducing apoptosis of growth plate chondrocytes^5^. Apoptosis is linked to high expression of caspase-3^6^, whereas PCNA^7^, and collagen X^8^ are decreased in conditions of growth retardation. In addition, Indian hedgehog also plays a crucial role in controlling proliferation and differentiation within the growth plate^9^. Systemically pro-inflammatory cytokines are known to suppress pituitary growth hormone (GH) release^10,11^. Nevertheless, it is unknown whether TNF overexpression can cause bone growth retardation, although it has been reported that anti-TNF treatment in JIA patients can rescue bone growth^12^. Similarly, studies in healthy mice have shown that GC treatment inhibits longitudinal bone growth by suppressing hedgehog-signaling, chondrocyte proliferation and differentiation while increasing cellular apoptosis^7,13,14^. However, it is still unknown how GCs affect bone growth in a disease model of chronic inflammation.

A limitation of previous experimental studies investigating GC effects on bone growth is that these have used healthy animal models and thereby provide no information on the effects in conditions of chronic inflammation. To address this, we used a model system of chronic inflammation induced by overexpression of human TNF in mice (huTNFTg). huTNFTg mice spontaneously develop arthritis and have previously been used to investigate the role of systemic TNF overexpression in different inflammation-driven pathologies^15–17^. Although TNF has been shown to suppress osteoblast activity and bone formation in young mice^18^, it is still unknown if TNF-overexpression may suppress growth plate chondrogenesis and longitudinal bone growth. To address this gap of knowledge, we characterized how growth plate chondrocytes and bone growth are affected in huTNFTg mice and how treatment with a GC, dexamethasone, modulates these effects.

## Materials and methods

### Animals

All animal experiments were performed at Biomedcode Hellas S.A. (Vari, Greece) and were approved by the BSRC Al. Fleming Institutional Committee of Protocol Evaluation in conjunction with the Veterinary Service Management of the Hellenic Republic Prefecture of Attica according to all current European and national legislation and were performed in accordance with relevant guidelines and regulations. The human TNF-over-expressing transgenic (huTNFTg, Tg197) mouse model (males and females after genotyping) described previously^15^ was used in this study. Briefly, the huTNFTg mouse line expresses increased levels of human TNF resulting in the spontaneous development of fast progressing arthritis, a cachectic phenotype and shortened lifespan. We also used untreated age-matched wild type (WT) female C57BL/6 mice to measure femur bone length and growth plate height*.* The number of animals per group were decided based on power calculations.

### Treatment and tissue sample collection and preparation

Starting from four weeks of age, the huTNFTg animals received daily subcutaneous injections with dexamethasone (3 mg/kg) or saline. The dose of dexamethasone was chosen based on a previous study from our group^7,15^. Mouse health was monitored daily. Body weight and arthritis scores were recorded on a weekly basis as previously described^19^. Briefly, arthritis score was evaluated in the ankle joints by clinical assessment using a semi-quantitative score ranging from 0 to 3 based on the severity of joint swelling and grip strength; 0 = no arthritis, 1 = mild arthritis, 2 = moderate arthritis, and 3 = severe arthritis. We included 22 huTNFTg in this study and divided them into 4 groups: group 1 (n = 5) was males treated with saline; group 2 (n = 5) was females treated with saline; group 3 (n = 6) was males treated with dexamethasone (3 mg/kg); group 4 (n = 6) was females treated with dexamethasone (3 mg/kg). We also had 6 age-matched wild type (WT) females C57BL/6 mice treated with saline. After 4 weeks of treatment, all animals were sacrificed with CO_2_. Hind limbs from huTNFTg animals and WT animals were dissected for histopathological analysis. Femur lengths were measured by digital caliper and thereafter the bone was fixed in 4% formaldehyde for 24 h followed by decalcification in EDTA buffer for 3–4 weeks before dehydration and paraffin embedding. All bone sections were sliced with the same orientation, placing controls and treatment groups on the same slide.

### Immunohistochemistry

To analyze protein expression in the growth plate, immunohistochemistry was performed in serial sections of femur growth plates with minor modifications to what has been described before^20^. Briefly, after deparaffinization and rehydration, antigen retrieval was performed in sodium citrate buffer (10 mM pH 6.0) for 15 min at 75 °C. After retrieval, the slides were blocked with 2% serum (horse and goat) followed by incubation overnight at 4 °C with primary antibody (1:100). Sections were incubated with anti-caspase 3 antibody (sc-1226; Santa Cruz Biotechnology, Dallas, TX, USA), anti-proliferating cell nuclear antigen (PCNA) antibody (ab-18197; Abcam, Cambridge, United Kingdom), anti-collagen X antibody (ab-58632 Abcam, Cambridge, United Kingdom) and anti-Ihh antibody (sc-1196; Santa Cruz Biotechnology). Sections were incubated for 1 h at room temperature with corresponding secondary antibody (1:300, BA-9500 Vector Laboratories; 1:500, ab 97,049 Abcam) followed by incubation with an avidin-peroxidase complex (Vectastain ABC-kit PK-6100) and visualized with 3,3' diaminobenzidine (DAB) (Dako K3468) development for 2–3 min. The slides were counterstained with Alcian Blue and dehydrated. Image J software (NIH) was used to quantify stained area and percentages of immunopositive cells in the growth plates.

### Histomorphometric analysis of growth plates

For histomorphometric analyses, serial sections (5 μm thick) of the femur growth plate were stained with hematoxylin–eosin which has been described before^7^. Images were photographed at 20 × magnification. Total growth plate height was measured as reported elsewhere^21^. Briefly, the measurement was performed from the proximal end of the resting zone to the chondro-osseous junction at the distal end of the growth plate. The height was measured at three different sites (medial, central, and lateral) of each growth plate and calculated as the average of three measurements. The height of the hypertrophic zone was measured in a similar way. The measurement was taken at the medial, central and lateral sites of the growth plate, from the first hypertrophic chondrocyte to the chondro-osseous junction, and the height was calculated as the average of three measurements. Hypertrophic cells were defined as cell more than 6 μm in height^22^. The number of hypertrophic cells were counted in the middle two-thirds of each growth plate and normalized by square millimeter. The size of hypertrophic cells was measured in 10 columns in each growth plate and calculated as the average of 10 measurements^22^. A chondrocyte column was defined as a column containing a minimum of 5 chondrocytes^23^. All measurements were done using the Image J Software by a person blinded to the experimental details.

### Statistics

Statistical significance of differences between groups was evaluated by 2-tailed Student’s t tests with 95% confidence intervals or Mann–Whitney Rank Sum Test. All data are expressed as mean ± SD or mean ± SEM. *P* values less than 0.05 were considered significant.

### Emirathics statement

This study was performed in Biomedcode's animal facility, that is an independent wing of BSRC Al. Fleming animal facility, registered by the Official Veterinary Service of the Prefecture of Attica as Breeding, Experimental and Supplying Laboratory Animal Facility, under the Registration Codes EL 09 BIO 04, EL 09 BIO 05 and EL 09 BIO 06, respectively. All of the conditions of testing conformed to the Presidential Decree No 56/2013 Governmental Gazette No A’ 106 applicable in Greece, which is the implementation of the EEC Directive2010/63/EEC and were approved by the directorate of Agricultural and Veterinary Policy (DAVP) of the Attica Region (Approval license 911/05/05/2015, a general experimental license covering academic experimental work including Tg197 mice). This in vivo study is in accordance with ARRIVE guidelines.

## Results

### Impaired bone growth and reduced growth plate height in huTNFTg mice

**Figure 1:**
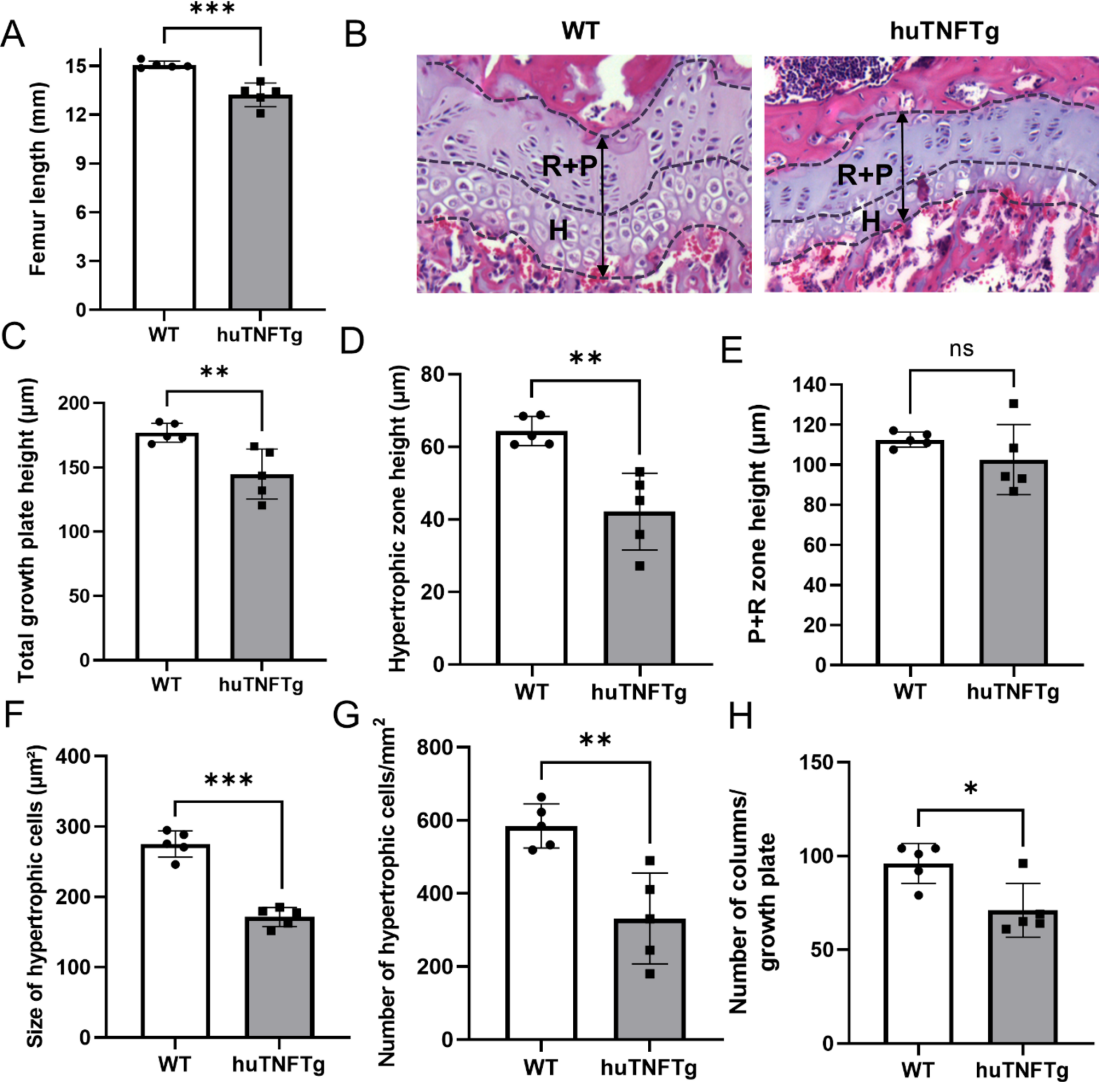
Overexpression of human TNF impaired bone growth and chondrogenesis in mice. Analysis performed in female wild type (WT) and female huTNFTg mice (n = 5). **(A)** Femur lenth in 8-week-old mice measeured at sacrifice by caliper. **(B)** Representative images indicating different zones and growth plate height in the femur in each group. **(C)** Total growth plate height. **(D-E)** Height measurements of hypertrophic zone and resting + proliferative (R + P) zone. (**F**) Measurements of size of hypertrophic cells in femur growth plates in each group. **(G)** Analysis of number of hypertrophic cells per growth plate section in each group. **(H)** Analysis of number of columns per growth plate section in each group. Error bar indicate mean ± SD. Students t-test was used to analyze differences between groups. *p < 0.001, **p < 0.01, ***p < 0.001.

We first measured femur length in 8-week-old female mice and found that the bone was shorter in huTNFTg animals when compared to wild type controls (*p* < 0.001) (Fig. 1A). Histomorphometric analysis of the growth plates showed that total growth plate height was decreased in huTNFTg animals compared to wild type (*p* < 0.01) (Fig. 1B,C). Similarly, the hypertrophic zone was also reduced in huTNFTg mice (*p* < 0.01 vs. wild type) (Fig. 1D). In contrast, the height of the resting + proliferative zone was not suppressed in huTNFTg animals (Fig. 1E). Both the size and number of chondrocytes were decreased in the hypertrophic growth plate zone of huTNFTg animals (*p* < 0.001 and *p* < 0.01 vs. wild type, respectively) (Fig. 1F,G). The number of chondrocyte columns per growth plate was also reduced in huTNFTg animals (*p* < 0.05 vs. wild type) (Fig. 1H).

## Suppressed chondrogenesis in huTNFTg mice

**Figure 2:**
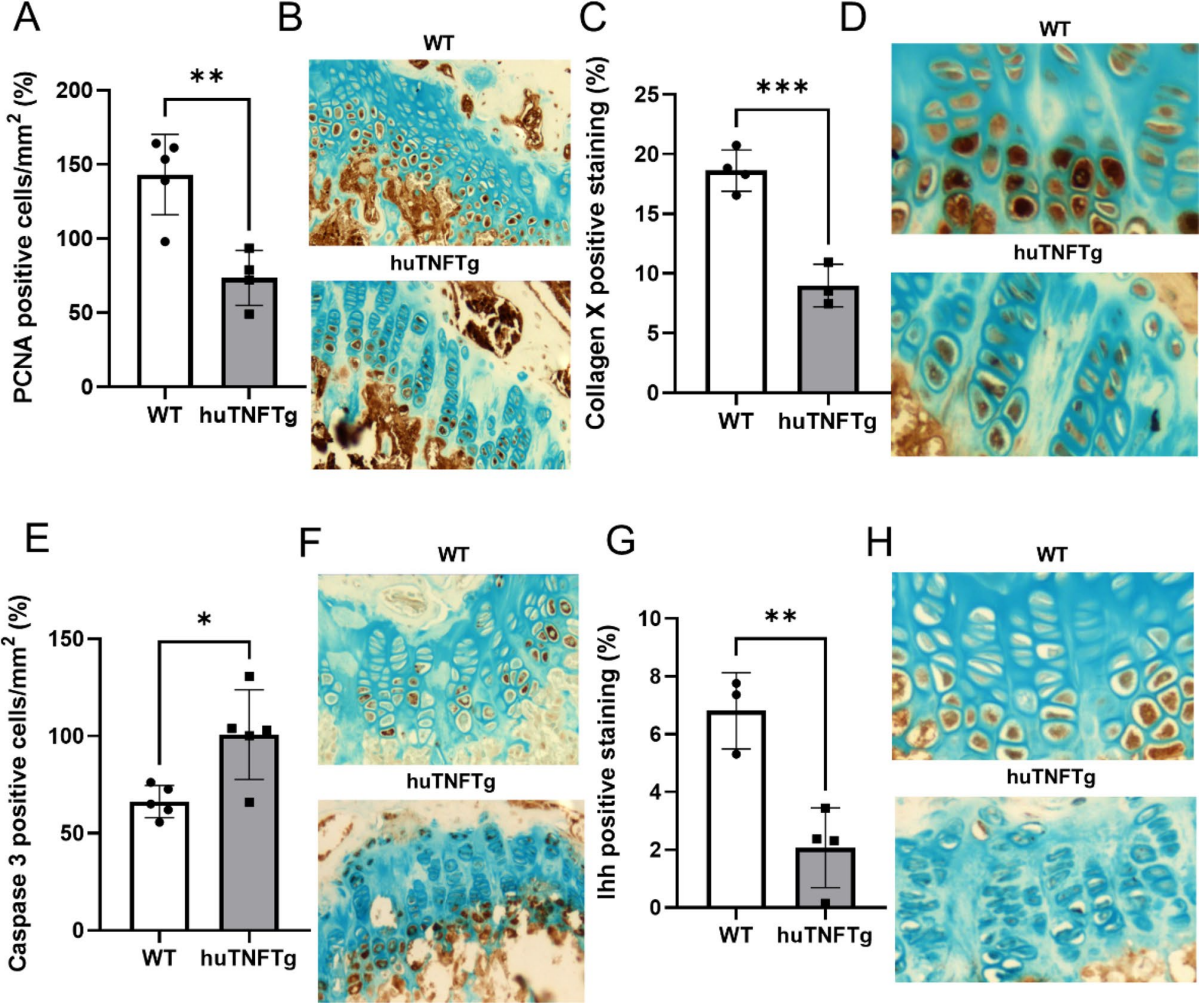
Local effects of human TNF overexpression on chondrocyte proliferation, apoptosis, hypertrophy and Ihh-cxprcssion. Immunohistochemistry analysis performed in femur growth plates of female wild type (WT) and female huTNFTg mice (n = 3–5). (**A, B**) Quantitative analysis of PCNA staining. (**C, D**) Quantitative analysis of collagen type-X staining. (**E, F**) Quantitative analysis of caspase-3 staining. (**G, H**) Quantitative analysis of Ihh staining. PCNA and caspase-3 staining quantified as number of positive cells per mm^2^. Ihh and collagen type-X staining calculated as percent of positively stained area over total growth plate area. Error bars indicate mean ± SD. Students t-test was used to analyze differences between groups. *p < 0.05, **p < 0.01 ***p < 0.001.

When further investigating growth plate chondrogenesis in huTNFTg animals, we first observed that cell proliferation, as measured by PCNA expression, was decreased by 48.7% (*p* < 0.01 vs. wild type; Fig. 2A,B). Chondrocyte hypertrophy, as measured by collagen X expression, was significantly suppressed in huTNFTg animals (*p* < 0.001 vs. wild type; Fig. 2C,D). In contrast, the apoptosis level, as measured by caspase-3 expression, was increased in huTNFTg animals (*p* < 0.05 vs. wild type; Fig. 2E,F). Since hedgehog-signaling plays a crucial role in controlling proliferation and differentiation within the growth plate cartilage, we next measured Indian hedgehog (Ihh) levels in the growth plates of huTNFTg animals and healthy wild type mice. The analysis revealed significant suppression by 69.6% of Ihh expression in huTNFTg mice (*p* < 0.01 vs. wild type; Fig. 2G,H).

### Effect of dexamethasone treatment on bone growth in huTNFTg mice

**Figure 3:**
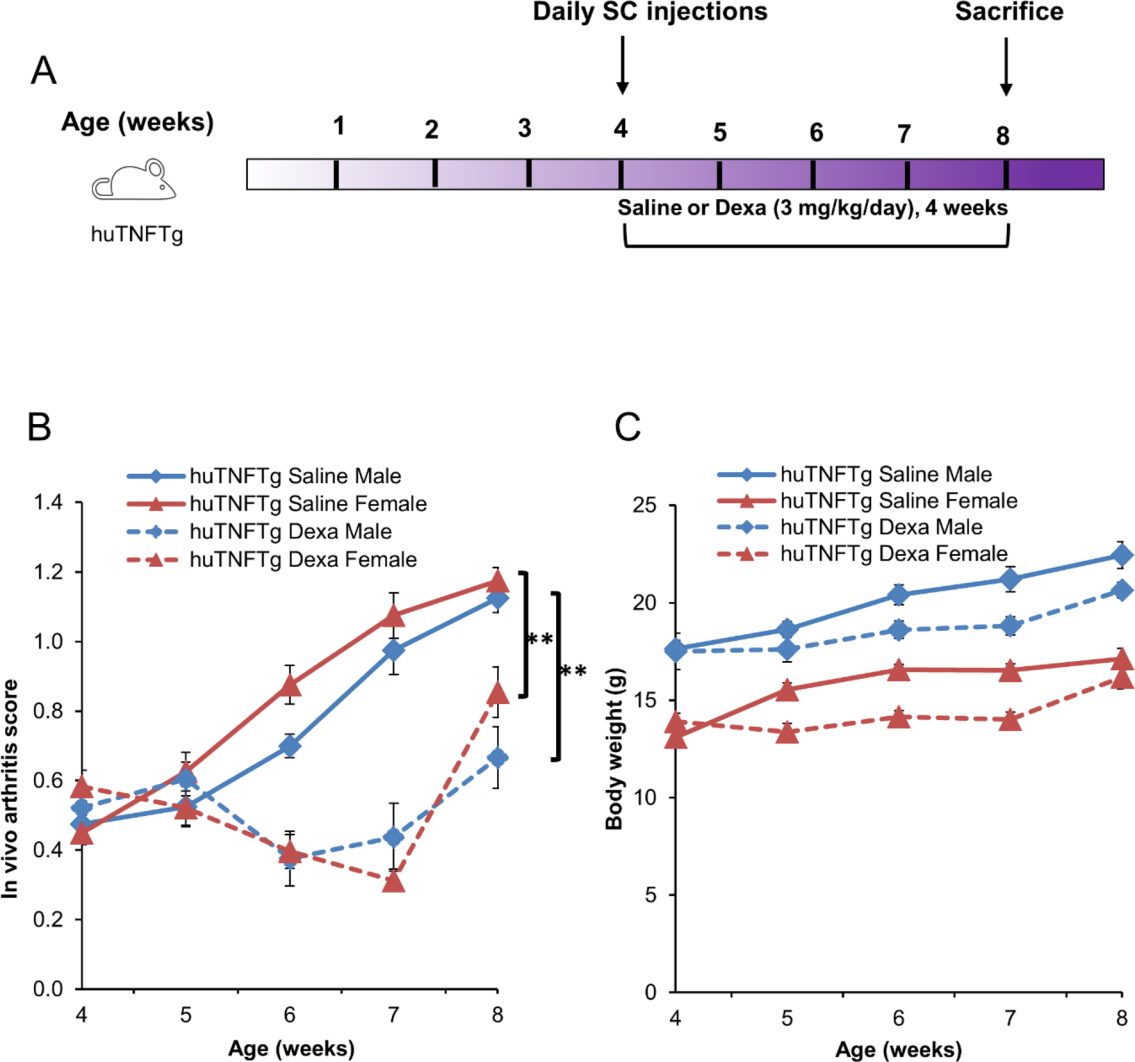
Effect of Dexamethasone (Dexa) on inflammation and body weight in huTNFTg mice (n = 5–6). (**A**) Schematic representation of experimental procedure, showing four-week-old huTNFTg mice treated with Dexa (3 mg/kg/day) or saline for 4 weeks. (**B**) Arthritis scores in male and female huTNFTg mice during 4 weeks treatment with saline or Dexa. (**C**) Body weight in male and female huTNFTg mice administered with Dexa or saline for 4 weeks. Error bars indicate mean ± SEM. Students t-test or Mann–Whitney Rank Sum test was used to analyze differences between groups. **p < 0.01.

**Figure 4:**
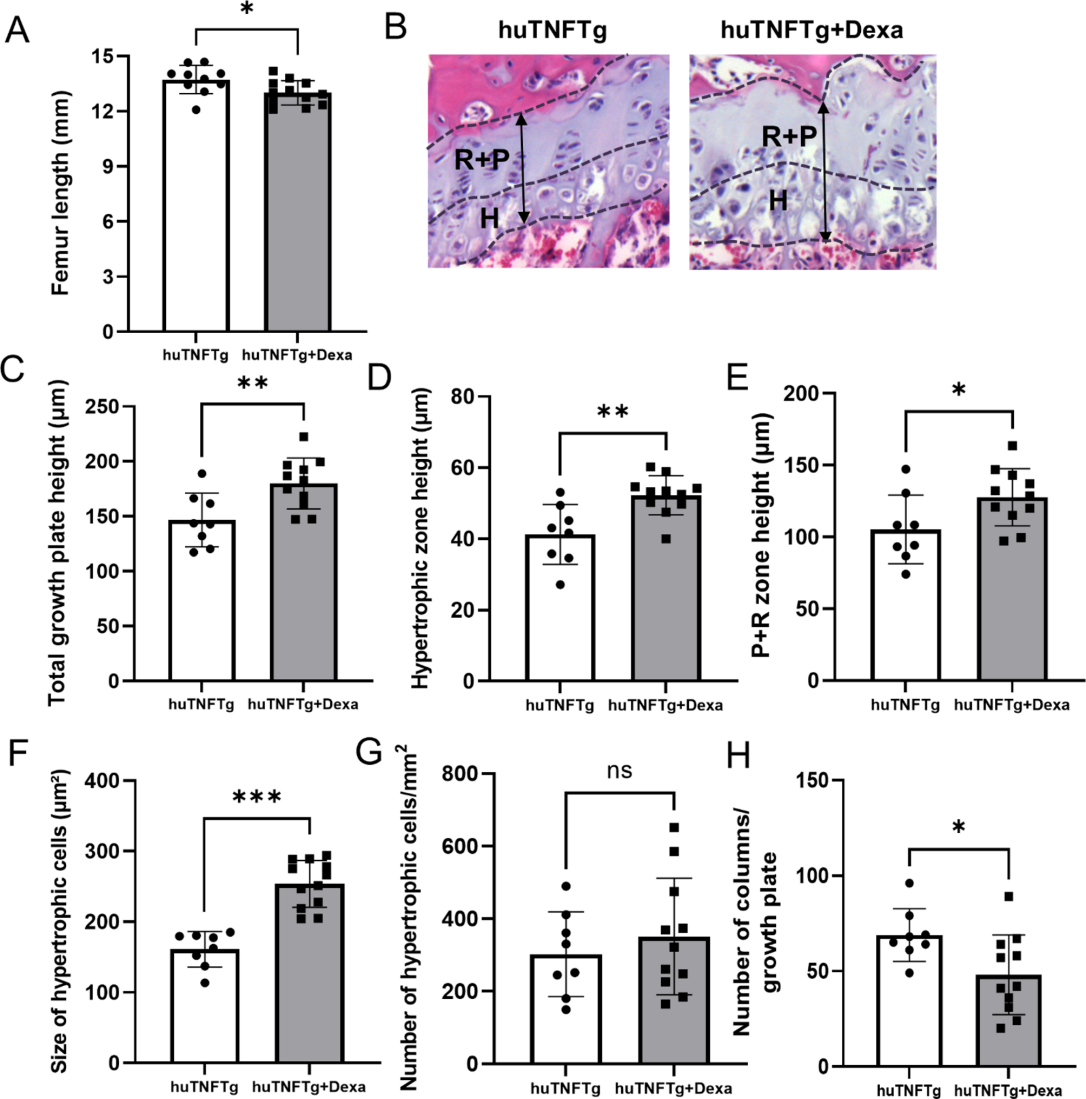
Effects of Dexamethasone (Dexa) treatment on bone growth and chondrogenesis in huTNFTg mice, combined males and females (n = 8–12). (**A**) Femur length measured by digital caliper. (**B**) Representative images indicating different zones and femur growth plate height in each group. (**C**) Measurements of total growth plate height, (**D, E**) the height of the hypertrophic zone and the resting + proliferative (R + P) zone, (**F**) and the mean size of hypertrophic cells in the femur growth plate in each group. (**G, H**) Analysis of the number of hypertrophic cells and the number of columns per growth plate in each group. Error bars indicate mean ± SD. Students t-test was used to analyze difference between groups. *p < 0.05, **p < 0.01, ***p < 0.001.

To determine whether GC treatment may affect bone growth in huTNFTg mice, a daily subcutaneous injection with dexamethasone (3 mg/kg body weight) or saline was administered for 4 weeks (Fig. 3A). As expected, the inflammatory activity was decreased in dexamethasone-treated huTNFTg mice as shown by lower arthritis scores, both in males and females, when compared to saline-treated huTNFTg controls (*p* < 0.01) (Fig. 3B) while body weight was only marginally suppressed by the dexamethasone treatment (Fig. 3C). In contrast, when combining male and female data femur length was significantly decreased in dexamethasone-treated huTNFTg mice (*p* < 0.05 vs. saline treated control) (Fig. 4A). Despite showing suppressed growth when combining males and females, the total growth plate height was significantly increased in the dexamethasone group when compared to saline-treated controls (*p* < 0.01) (Fig. 4B,C). Similarly, the heights of the hypertrophic zone and the combined proliferative + hypertrophic zone were both increased in the dexamethasone group (*p* < 0.01 and *p* < 0.05 vs. saline treated controls, respectively) (Fig. 4D,E). Histomorphometric analysis showed that the mean size of the hypertrophic chondrocytes was increased by 36.5% in dexamethasone treated huTNFTg mice (*p* < 0.001 vs. saline-treated control) (Fig. 4F), whereas the number of hypertrophic chondrocytes did not differ between the two groups (Fig. 4G). However, the number of chondrocyte columns was significantly reduced by the dexamethasone treatment (*p* < 0.05) (Fig. 4H).

**Figure 5:**
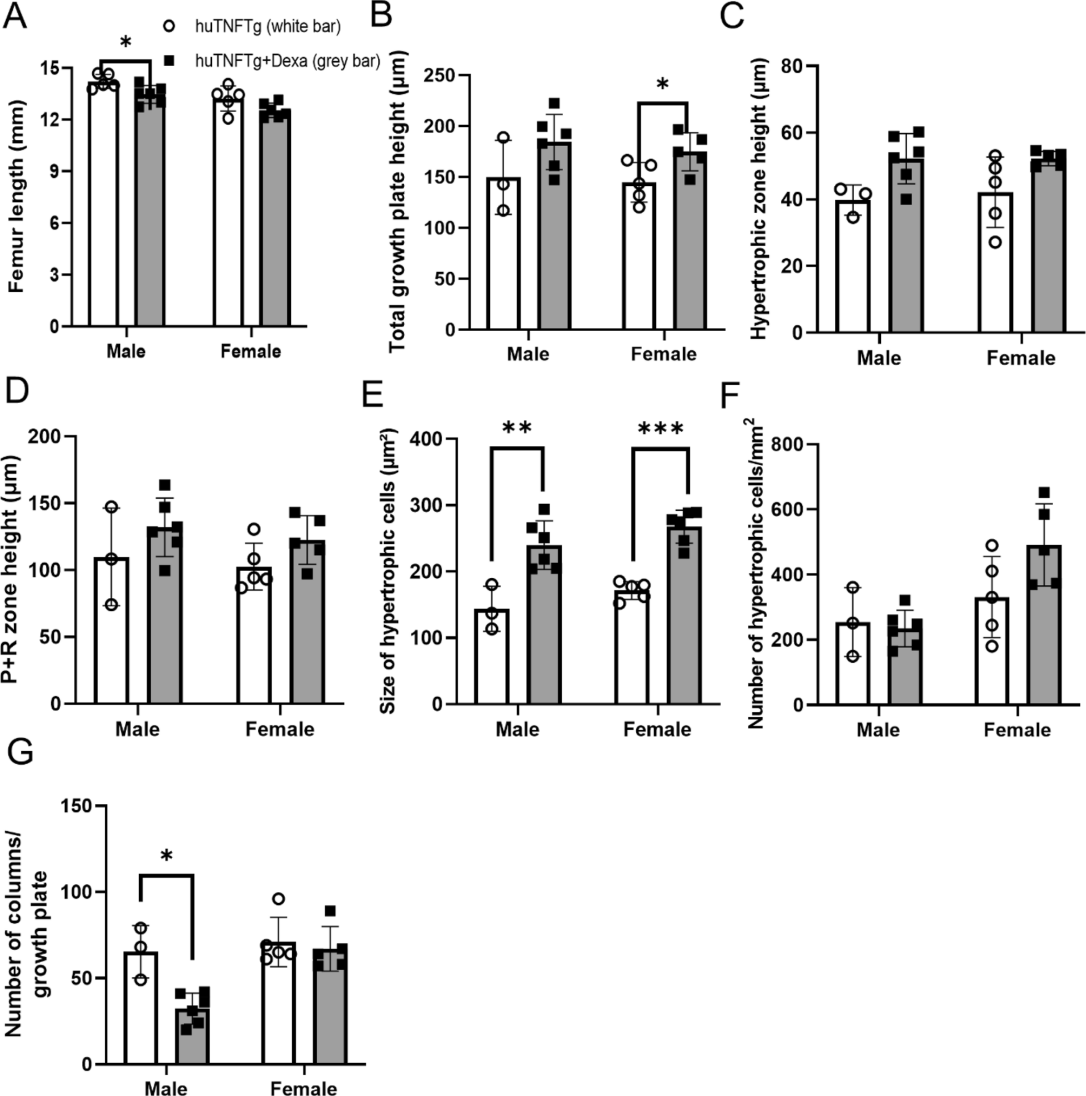
Effects of Dexamethasone (Dexa) treatment on bone growth and chondrogenesis in male and female huTNFTg mice. (**A**) Femur length measured by digital caliper (n = 5–6). (**B**) Total growth plate height, (**C**) height of the hypertrophic zone, and (**D**) height of the resting + proliferative (R + P) zone in femur growth plates in each group. (**E**) Measurements of the size of hypertrophic cells in femur growth plates in each group. (**F**) Analysis of number of hypertrophic cells per growth plate (**G**) and number of columns per growth plate in each group (n = 3–6). Error bars indicate mean ± SD. Students t-test was used to analyze difference between groups. *p < 0.05, **p < 0.01, ***p < 0.001.

When analyzing males and females separately, dexamethasone only significantly decreased femur length in males (Fig. 5A). The total growth plate height was increased in females but not in males treated with dexamethasone (Fig. 5B). The heights of the hypertrophic zone, and the combined proliferative + hypertrophic zone were not affected by dexamethasone treatment in neither males nor females (Fig. 5C,D). Dexamethasone treatment increased the mean size of the hypertrophic chondrocytes in both males and females (Fig. 5E) without affecting the number of hypertrophic chondrocytes (Fig. 5F), whereas the number of chondrocyte columns was decreased in males treated with dexamethasone (Fig. 5G).

### Effects of dexamethasone treatment on chondrogenesis in huTNFTg mice

**Figure 6:**
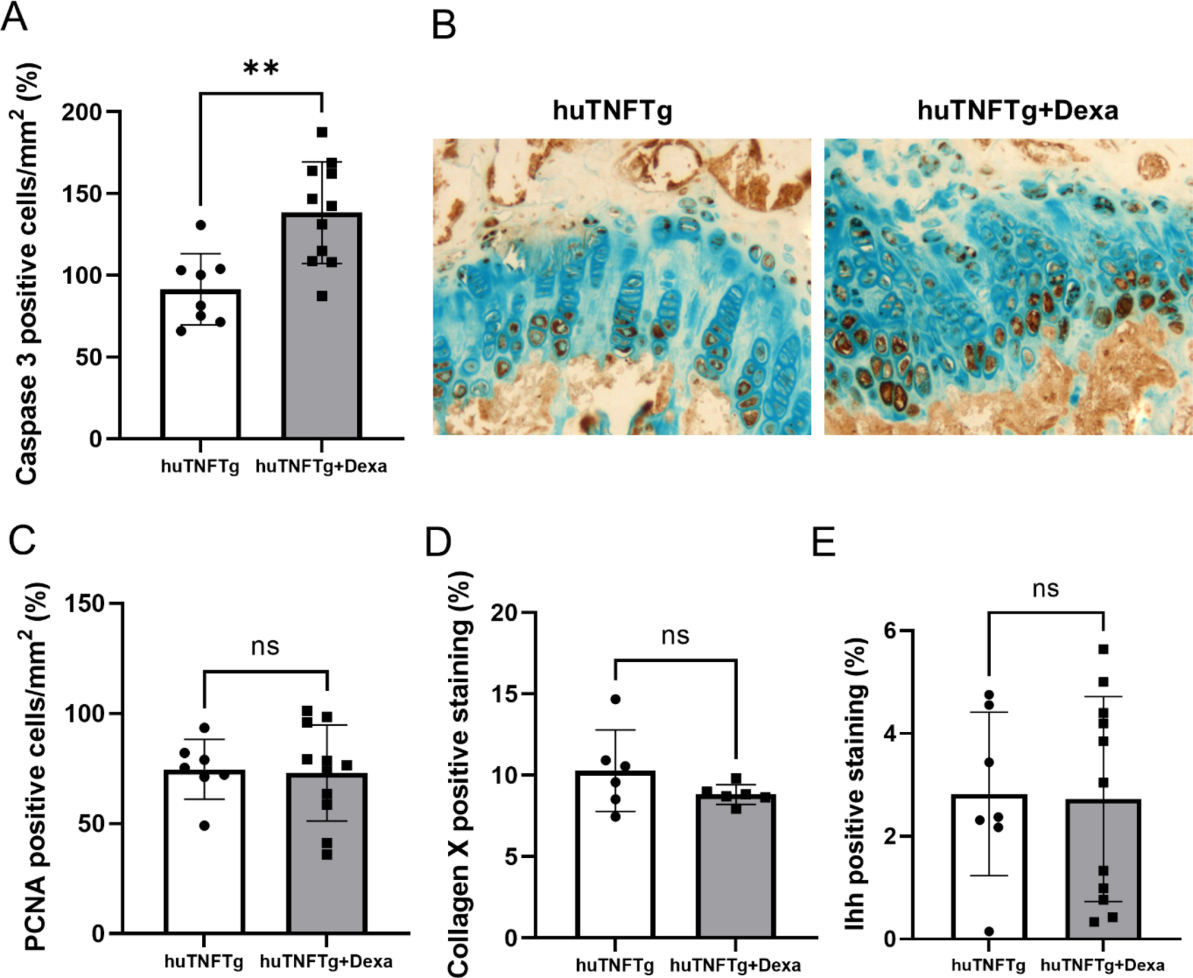
Effects of Dexamethasone (Dexa) treatment on chondrocyte proliferation, hypertrophy, Ihh-expression and apoptosis in huTNFTg mice, combined males and females (n = 6–11). (**A, B**) Quantitative analysis of caspase-3 staining and (**C**) PCNA staining, expressed as number of positive cells per mm^2^, (**D**) collagen X and (**E**) Ihh staining expressed as percent of positively stained area over total growth plate area. Error bars indicate mean ± SD. Students t-test was used to analyze differences between groups. **p < 0.01.

**Figure 7:**
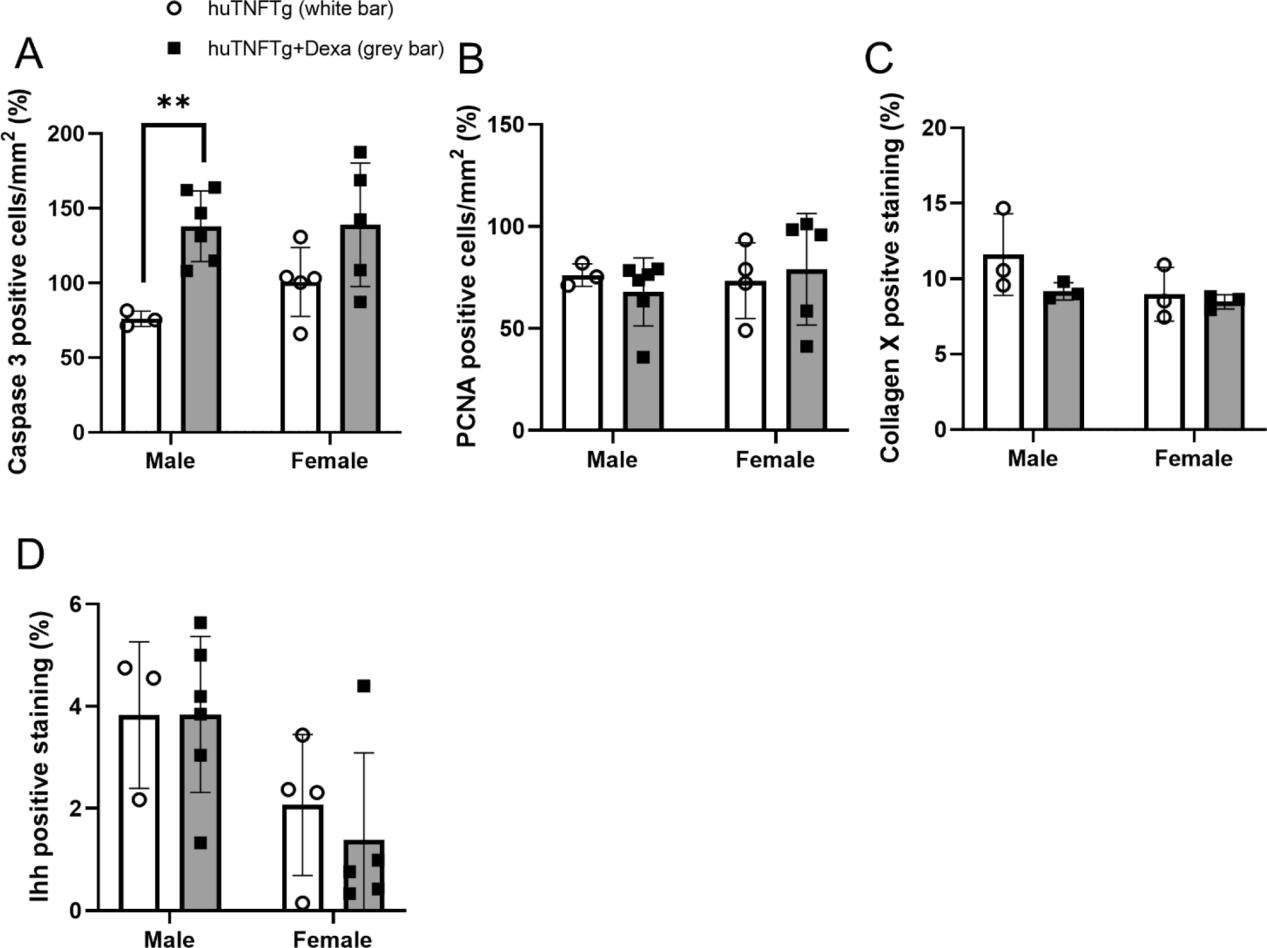
Effects of Dexamethasone (Dexa) treatment on chondrocyte proliferation, hypertrophy and apoptosis in huTNFTg mice (males and females analyzed separately). (**A**) Quantitative analysis of Ihh staining, (**B**) collagen type-X staining, expressed as number of positive cells per mm^2^, (**C**) PCNA staining, (**D**) and caspase-3 staining, expressed as percent of positively stained area over total growth plate area. (n = 3–6). **p < 0.01.

To investigate the underlying mechanisms of dexamethasone-induced growth impairment in huTNFTg mice, the expression levels of the proliferative marker PCNA, the hypertrophic marker collagen X, and the apoptosis marker caspase-3 were analyzed in the growth plates of male and female huTNFTg mice treated with dexamethasone or saline. Notably, dexamethasone treatment increased the expression level of caspase-3 (*p* < 0.01 vs. saline-treated control) (Fig. 6A,B), while the expression levels of PCNA, collagen X, and Ihh were not significantly affected (Fig. 6C–E). When separating males and females, we noticed that dexamethasone treatment increased caspase-3 expression in males only (Fig. 7A), whereas the levels of PCNA, collagen X and Ihh were not changed (Fig. 7B–D).

## Discussion

We here present novel data clarifying the individual roles of GC treatment and/or inflammation on bone growth in a mouse model of chronic arthritis where human TNF is overexpressed (huTNFTg). In huTNFTg animals, bone growth and growth plate chondrogenesis were found to be suppressed compared to wild type controls. When treating huTNFTg mice with dexamethasone, although an anti-inflammatory effect was documented, bone growth was further impaired. Mechanistic studies showed that the dexamethasone treatment further increased chondrocyte apoptosis and decreased the number of chondrocyte columns when compared to untreated huTNFTg controls.

To our knowledge, this is the first in vivo characterization of the effects of TNF-overexpression on bone growth and chondrogenesis. Although overexpression of TNF in mice has been associated with decreased body size^24,25^ and bone formation^18^, any effect on longitudinal bone growth and growth plate chondrogenesis has not yet been reported. Our primary finding was that bone growth was impaired in young mice exposed to increased TNF-levels throughout development. Although TNF has been earlier described to suppress the growth of ex vivo cultured fetal rat metatarsal bones, this effect was only achieved at a very high concentration^11^.

We report that overexpression of TNF in mice not only suppressed bone growth, but detailed histomorphometry also showed reduced growth plate height where the hypertrophic zone was most affected. Besides reduced hypertrophic zone height, we also observed decreased size and number of hypertrophic chondrocytes suggesting that within the growth plate, these cells were most affected when overexpressing human TNF. We also report that chondrocyte proliferation was suppressed in huTNFTg mice. Since hedgehog-signaling is known to play a crucial role in controlling proliferation and differentiation within the growth plate cartilage, expression levels of Indian hedgehog (Ihh) were analyzed. We then found that the expression of Ihh was significantly decreased in the growth plate cartilage of the huTNFTg animals when compared to wild type controls. In a diabetic mouse model, elevated levels of TNF have previously been shown to directly suppress Ihh expression in skeletal stem cells suggesting a link between TNF and Ihh signaling which is in line of our present findings in huTNFTg mice^26^. Furthermore, disruption of Ihh signaling in Ellis-van-Creveld syndrome protein knockout mice has been shown to not only cause limb shortening but also reduced height of the proliferative and hypertrophic growth plate zones^27^. We report that chondrocyte columns were fewer and disorganized in the TNF-overexpressing mice were the expression of Ihh was also suppressed. A similar disorganized growth plate structure was earlier reported in mice with disrupted Ihh signaling which further supports a link between TNF and Ihh signaling in the growth plate cartilage^27^.

To explore if an anti-inflammatory treatment with a glucocorticoid (GC) may rescue bone growth and chondrogenesis in an in vivo model of chronic arthritis, we also treated young huTNFTg mice with daily injections of dexamethasone or saline. We confirmed that dexamethasone not only suppressed the chronic arthritis but also restored the height of the total growth plate and the hypertrophic zone. However, despite these positive effects of the dexamethasone treatment, bone growth was further decreased compared to saline-treated huTNFTg controls. Interestingly, the observed increase in growth plate height in our study resembles the growth plate phenotype seen in hypophosphatemia^28^. The increased width of the growth plate has also been reported in conditions not associated with hypophosphatemia, such as abnormal IGF-1 metabolism^29^. Previously, it has been shown that glucocorticoids can not only impair GH-IGF-1 signaling^30^, but also cause hypophosphatemia in hospitalized patients^31^.

To clarify the underlying mechanisms for this unexpected finding, growth plate histomorphometry and immunohistochemistry was performed. We then found that despite successfully reducing inflammation, as verified by reduced arthritis score, the dexamethasone treatment was not able to restore chondrocyte proliferation in the growth plates of huTNFTg mice. Strikingly, the high apoptotic activity and disorganized chondrocyte columns observed in huTNFTg mice were further enhanced by the dexamethasone treatment. We therefore conclude that the anti-inflammatory effect of dexamethasone could not compensate for the negative effect of the GC treatment on growth plate. Instead the combination of TNF and dexamethasone further worsened longitudinal bone growth. A link between disorganized chondrocyte columns in the growth plate and impaired bone growth has earlier been reported in mice^27^. Furthermore, dexamethasone treatment has been reported to suppress longitudinal bone growth by increasing chondrocyte apoptosis in the growth plates of treated mice^7,13^. These previous reports support our conclusion that increased chondrocyte apoptosis and disorganized chondrocyte columns may, at least partly, explain how dexamethasone further impaired bone growth despite suppressing the chronic arthritis in the huTNFTg mice. Importantly, body weight was not significantly affected in the young huTNFTg mice treated with dexamethasone suggesting that the observed growth suppressive effect of dexamethasone was not linked to poor general health.

We observed that although the caspase-3 levels were increased after dexamethasone treatment, the growth plate height, including the hypertrophy zone, was increased, despite bone growth was impaired. A similar observation was earlier reported in a mouse model of osteogenesis imperfecta^32^, where growth plate height was increased despite bone length was decreased. Interestingly, it has also been shown that apoptosis is increased in the growth plates of mice with osteogenesis imperfecta, which potentially contributes to the reduction of the mineralized area in the hypertrophic zone^33^. These observations suggest similarities between dexamethasone induced bone growth suppression and growth impairment as seen in osteogenesis imperfecta, because in both conditions apoptosis is increased while growth plate height is also increased. Further, increased apoptosis in osteoblasts was observed in osteogenesis imperfecta where longitudinal bone growth is often decreased^34^. Similarly, GCs are known to increase apoptosis in osteoblasts^35^, which suggests that osteoblasts might also play a role in GCs induced growth retardation under chronic inflammation. However, more experimental evidence is required to address this complex question in the future. Another explanation might be that caspase-3 is not only related to cell death, but also to hypertrophy. It has been previously reported that activation of caspase-3 plays a key role in cardiac hypertrophy^36^. Similarly, another study showed a strong link between hypertrophy and caspase-3 expression in epiphyseal chondrocytes^37^. Altogether, the complex cross-talk between bone growth and caspase-3 expression and apoptosis needs to be further investigated.

There are several potential limitations in our study. First, despite TNF is one of the predominant cytokines in arthritis, there might be other pro-inflammatory cytokines upregulated as part of the cytokine response. We have previously shown that TNF and IL-1β act in synergy to cause growth retardation in cultured ex vivo fetal rat metatarsal bones^38^. Also, the expression of IL-6 has been reported to be increased by TNF through NF-κB^39,40^. As both IL-6 and IL-1β have been described to be upregulated in TNF-overexpressing mice^41^, these pro-inflammatory cytokines may have contributed to the bone growth suppression and impaired chondrogenesis here reported in huTNFTg mice. A second limitation is the fact that bone length measurements and mechanistic studies were only performed at endpoint and therefore the timing of the different effects could not be further detailed. A third limitation is the relative low number of animals per group which makes sub-analyses of any sex differences difficult.

In summary, this study for the first time shows how TNF and dexamethasone impair bone growth separately, and in an additive manner. TNF-driven chronic inflammation in mice is linked to suppressed chondrocyte proliferation and hypertrophy as well as increased apoptosis and disorganized chondrocyte columns in the growth plate cartilage. Dexamethasone treatment, despite efficiently suppressing the inflammatory activity, unexpectedly accelerated chondrocyte apoptosis and caused chondrocyte columns to become more disorganized in the growth plates and further suppressed bone growth. These results give a possible explanation to how GC treatment may further impair bone growth in children with chronic inflammatory conditions. When developing new effective treatments of chronic inflammatory conditions, our data highlights the importance of choosing therapeutic strategies with minimal effects on the growth plate cartilage thereby preventing bone growth impairment.

## Research in context

### Evidence before this study

Children with chronic inflammatory disorders are often treated with glucocorticoids and many experience growth retardation where the underlying mechanisms are not fully understood. In preclinical models, proinflammatory cytokines and glucocorticoids have individually been shown to impair bone growth, but how they interact is still unclear.

### Added value of this study

This study supports a link between systemic inflammation and impaired bone growth in a mouse model of chronic arthritis where human TNF is overexpressed (huTNFTg). The data presented suggests that glucocorticoids, despite having an anti-inflammatory activity, further impair bone growth and chondrogenesis in huTNFTg mice. The study also provides mechanistic data showing that glucocorticoid treatment may further trigger chondrocyte apoptosis and loss of chondrocyte columns in the growth plates of huTNFTg mice.

### Implications of all the available evidence

Our data provide a better understanding of possible mechanisms behind the growth retardation commonly observed in children with chronic inflammatory diseases. Our experimental data suggest that glucocorticoids may further impair bone growth and chondrogenesis when used to treat a chronic inflammatory condition. This study emphasizes the need of new anti-inflammatory treatment strategies that do not cause undesired apoptosis in the growth plate, which should reduce the risk of growth failure in children.

## Data Availability

Study data are available from the corresponding author upon reasonable request.
